# Terbinafine-Induced Hepatitis B Reactivation in a Patient With Chronic Hepatitis B

**DOI:** 10.7759/cureus.11958

**Published:** 2020-12-07

**Authors:** Elona Shehi, Ahmed Alemam, Nikhitha Mantri, Harish Patel

**Affiliations:** 1 Gastroenterology, BronxCare Health System, Bronx, USA; 2 Internal Medicine, BronxCare Health System, Bronx, USA

**Keywords:** terbinafine, hepatitis b, flare, transaminitis

## Abstract

Reactivation of chronic inactive hepatitis B infection can be spontaneous or triggered by chemotherapy or immunosuppression. Although the pathophysiology behind this remains unclear, several mechanisms have been proposed in the literature. Data describing hepatitis B flares caused by other classes of medications are sparse. We describe a case of reactivation of chronic hepatitis B in a 48-year-old man, who presented with generalized weakness after initiating terbinafine for a fungal toenail infection. He was found to have deranged liver function tests and elevated hepatitis B viral load. Both resolved after discontinuation of terbinafine and starting tenofovir.

## Introduction

Drug-induced hepatitis is a well-known clinical entity; however, reactivation of a previously chronic inactive viral hepatitis caused by medications does not have similar awareness among clinicians and can often be overlooked. Screening for hepatitis B infection and closely monitoring for hepatitis B reactivation is recommended in patients planned for chemotherapy or immune-suppressive therapy [1]. Serologic tests include hepatitis B surface antigen (HBsAg), hepatitis B core antibody (anti-HBc), and hepatitis B DNA viral load. Hepatitis B reactivation can be asymptomatic, manifesting only as serologic evidence of flare, or can present with symptoms of weakness, fatigue, nausea, and malaise. Hepatitis B reactivation has been described in patients receiving the biological therapy for the inflammatory bowel disease [2], chemotherapy for solid and hematological malignancy [3,4] and those receiving chemoembolization for hepatocellular carcinoma [5]. Hepatitis B relapse is common after discontinuation of the nucleic acid analogs [6] and rarely can lead to fatal hepatitis B reactivation [7]. Terbinafine is an orally and topically active anti-fungal agent commonly used to treat superficial skin and nail infections, although the drug is known to cause multiple side effects commonly involving the gastrointestinal system like nausea, vomiting, dyspepsia, diarrhea, and rarely hepatic injury. Terbinafine has been reported to cause autoimmune hepatitis in a patient with chronic hepatitis B infection [8], but there is no available literature on terbinafine causing hepatitis B reactivation. We present a case of a patient developing an acute flare of hepatitis B infection after he was started on terbinafine for a fungal toe infection.

## Case presentation

A 48-year-old African man with medical comorbidities of chronic hepatitis B was referred to our infectious disease clinic for hepatitis B management. The patient was born and raised in Ghana and moved to the United States 12 years ago. He was a non-smoker and denied using any alcohol or recreational drugs. He was not sexually active and worked as a security guard. Family history was positive for chronic hepatitis B in the mother. At the time of presentation, on physical examination patient was well appearing, skin exam showed no evidence of rash, icterus, spider angiomatas, palmar erythema, and no visible varicose veins. Cardiovascular and pulmonary exams were unremarkable, abdominal exam showed no organomegaly, absence of ascites, no tenderness on superficial or deep palpation. His laboratory were significant for normal liver function tests: aspartate aminotransferase (AST): 32 units/L and alanine aminotransferase (ALT): 36 units/L, albumin 5.2 g/dl, alkaline phosphatase (ALP) 119 units/l, total bilirubin 0.7 mg/dl and direct bilirubin 0.2 mg/dl. Other laboratories: HIV and hepatitis C virus (HCV) testing were negative, hepatitis A virus (HAV) antibodies positive, HBsAg positive, HBsAb negative, hepatitis B core antibody (HBcAb) total-positive, and hepatitis B viral load of 19,091 IU/L (normal: <20 IU/L).

He was started on emtricitabine and tenofovir combination (Truvada) for the treatment of hepatitis B, but shortly after starting treatment, he was lost to follow up. Ten months later, he presented to his primary care physician's office with what appeared to be a fungal toenail infection. During that encounter, he admitted that he stopped Truvada one month after it was initially prescribed. Labs done at that time showed normal liver function tests and viral load in the previous range. He was prescribed terbinafine 250 mg orally once a day for 12 weeks. One month after starting terbinafine, the patient presented to his primary care physician's office with complaints of generalized weakness and muscle aches of two weeks duration. The physical exam was unremarkable. As part of the basic workup, labs were done and were significant for transaminitis with AST of 294 u/l and ALT of 428 u/l. He denied any recent surgeries, blood transfusions, or dental work. He denied the use of any medications, herbal supplements, or intravenous drug use. The patient was referred to the hepatology clinic. He had repeated liver function tests which showed worsening transaminitis with AST of 601u/l and ALT of 943 u/l. The rest of his laboratory tests were: albumin 4.5 g/dl, alkaline phosphatase 165 unit/l, total bilirubin 1.3 mg/dl, direct bilirubin 0.7 mg/dl. Hepatitis B viral load was increased to 47 million copies. HBcIgM was negative. HIV and hepatitis C, A, D testing were negative. Anti-mitochondrial antibody and anti-smooth muscle antibody screening were negative. Lamisil (terbinafine) was stopped. He was started on tenofovir and two months later, his transaminitis resolved and hepatitis B viral load decreased to 3731 IU/ml.

## Discussion

Terbinafine, first introduced in 1998, has been associated with multiple cases of transaminitis and idiosyncratic liver injury. Clinically apparent liver injury from terbinafine occurs rarely (1 in 50,000 to 120,000 prescriptions). Abnormal liver chemistries usually arise within the first six weeks of therapy and resolve within three to six months after stopping the medication. The pattern of injury can be either hepatocellular or cholestatic initially, and typically evolves into a cholestatic pattern which can be prolonged and in some cases may progress to vanishing bile duct syndrome [9]. Signs of hypersensitivity (rash, fever, eosinophilia) are not common and, when present, are generally mild-to-moderate in severity [10]. Terbinafine is metabolized by the liver CYP (cytochrome P450) enzymes, and one of its metabolites 7,7-dimethylhept-2-ene-4-ynal (TBF-4), the allelic aldehyde metabolite, is believed to play a role in the pathogenesis of hepatotoxicity [11]. Another proposed mechanism of liver injury is through the activation of pro-inflammatory cytokines. Mizuno et al. proposed that terbinafine can stimulate monocytes and increases the release of IL-8 and tumor necrosis factor-alpha (TNF-alpha) [12].

To the best of our knowledge terbinafine has not been linked to hepatitis B or any other viral hepatitis reactivation. Reactivation of hepatitis B is a syndrome characterized by the abrupt appearance or rise of hepatitis B virus (HBV) DNA in the serum of a patient with previously inactive or resolved hepatitis B infection. At the initial encounter and prior to starting terbinafine, our patient was in the chronic inactive phase: he had normal aminotransferases levels, HbeAg negative, and HBV DNA < 20.000 IU/ml. One month after starting terbinafine, he presented with symptoms of fatigue, low energy, and muscle aches, and his laboratory tests were significant for elevated transaminases more than 10 times the upper level of normal, with a hepatocellular pattern of injury. Hepatitis B viral load was reported to be more than 40 million IU/ ml. Drug-induced liver injury from terbinafine was suspected, but the pattern of injury was hepatocellular and did not evolve into a cholestatic pattern, and the patient did not have any signs of hypersensitivity. Autoimmune hepatitis and other viral hepatitis were also excluded as a possible cause of transaminitis. The clinical presentation was consistent with hepatitis B reactivation, and terbinafine was suspected to be the possible trigger. Elevated HBV DNA levels are believed to be present in 40% of inactive carriers [12].

Reactivation of hepatitis B can be spontaneous, but it is mostly triggered by immunosuppression, chemotherapy, or alteration in the immune system [13]. Reactivation of hepatitis B is also reported after discontinuation of nucleoside analogs like lamivudine [7], but no cases of hepatitis B reactivation after stopping tenofovir are reported in the literature. The exact mechanism of hepatitis B reactivation is not known, but we propose that terbinafine promotes hepatitis B viral replication as a possible mechanism of reactivation. Mizuno et al. in their study found that terbinafine stimulates the release of pro-inflammatory cytokines: IL-8 and TNF-alpha [12]. Yu et al. studied in detail the role of inflammatory cytokines in hepatitis B replication and liver injury [14]. It was proposed that hepatitis B induces a regulatory loop of inflammatory cytokine network, in which three inflammatory cytokines IL-29, IL-8, and COX2 play a major role. IL-29 is able to inhibit the replication of a number of viruses, including HCV, and HBV. IL-8 may be a key mediator in the cytokine network induced by HBV. IL-8 not only impairs the antiviral activity of IL-29 and favors the establishment of persistent viral infection, but also induces the high expression of the pro-inflammatory factor COX-2 to maintain the inflammatory environment associated with HBV infection, playing so a major role in hepatitis B replication [14]. Spontaneous reactivation of hepatitis B cannot be fully excluded in our patient, but given that the time frame of the reactivation coincides with the time terbinafine was started, we believe that terbinafine was the culprit.

## Conclusions

Drug-induced liver injury is common and terbinafine related cholestatic hepatic injury is well reported. However, in patients with hepatitis B, terbinafine use can lead to hepatitis B reactivation. Though there is no prior data, we hypothesize that hepatitis B reactivation could due to terbinafine-related immune dyspepsia-regulation. Usage of terbinafine use should be cautious and careful monitoring is needed in patients with chronic inactive hepatitis B.

## Appendices

**Table 1:** Laboratory tests

	Day 0 (Time of initial presentation)	Month 10 - Day 1 (Second presentation)	Month 10 - Day 21	Month 10 - Day 27	Month 13	Month 17
Total protein (g/dl)	8.1	7.2	7.1	7.4	7.2	7.2
Albumin(g/dl)	5.2	4.5	4.5	4.7	4.9	4.7
AST (unit/L)	32	294	601	147	32	23
ALT (unit/L)	36	428	943	412	27	15
ALP (unit/L)	114	130	165	141	109	106
Total bilirubin (mg/dl)	0.7	0.8	1.3	1.3	0.5	0.6
Direct bilirubin (mg/dl)	0.4	0.3	0.7	0.4	0.2	0.2
Hepatitis A total Ab	Positive	-	-	-	-	-
Hepatitis B core total Ab	Positive	-	-	-	-	-
Hepatitis B core IgM Ab	-	-	-	Negative	-	-
Hepatitis B surface Ab	Negative	-	-	-	-	-
Hepatitis B surface Ag	Positive	-	-	-	-	-
Hepatitis B e Ab	-	Reactive	-	-	-	-
Hepatitis B e Ag	-	Non-reactive	-	-	-	-
Hepatitis D total Ab	-	-	-	Negative	-	-
Hepatitis B viral DNA cop/ml	4.28	7.68	-	-	3.57	2.9
Hepatitis B viral DNA IU/ml	19091	47933885	-	-	3731	800
Hepatitis C Ab	Negative	Negative	-	-	-	-
HIV (1/2 ab EIA)	Negative	Negative	-	-	-	-
Smooth muscle Ab screen	-	-	-	Negative	-	-
